# New Orleans Medical Times

**Published:** 1861-04

**Authors:** 


**New Orleans Medical Times.**

This is the title of the new Journal, published in New Orleans, in place of the New Orleans Medical News and Hospital Gazette, which expired with its February issue. Anthony Peniston, M. D., the editor of the new or metamorphosed Journal, we think, brings to the chair editorial, an amount of talent and energy quite sufficient to warrant us in looking for a continuance of the success which crowned the course of its predecessor, under the editorial control of D. Warren Brickell, M. D.

We welcome the "Times"—the March number of which has been received among our exchanges—and hope to draw upon it often, in making material for the pages of our Journal.

Dr. Brickell, since doffing the honors and responsibilities connected with the editorial management of the News and Gazette, has taken upon himself labors no less arduous, as editor of an annual publication, devoted to obstetrical subjects. We publish his prospectus on our advertising page of this number.

